# BIFNOM: Binary-Coded Features on Normal Maps

**DOI:** 10.3390/s21103469

**Published:** 2021-05-16

**Authors:** Leo Miyashita, Akihiro Nakamura, Takuto Odagawa, Masatoshi Ishikawa

**Affiliations:** Data Science Research Division, Information Technology Center, The University of Tokyo, Tokyo 133-8656, Japan; Akihiro_Nakamura@ipc.i.u-tokyo.ac.jp (A.N.); tk55-1234@g.ecc.u-tokyo.ac.jp (T.O.); ishikawa@ishikawa-vision.org (M.I.)

**Keywords:** surface normal, feature point, binary, real-time tracking

## Abstract

We propose a novel method for detecting features on normal maps and describing binary features, called BIFNOM, which is three-dimensionally rotation invariant and detects and matches interest points at high speed regardless of whether a target is textured or textureless and rigid or non-rigid. Conventional methods of detecting features on normal maps can also be applied to textureless targets, in contrast with features on luminance images; however, they cannot deal with three-dimensional rotation between each pair of corresponding interest points due to the definition of orientation, or they have difficulty achieving fast detection and matching due to a heavy-weight descriptor. We addressed these issues by introducing a three dimensional local coordinate system and converting a normal vector to a binary code, and achieved more than 750fps real-time feature detection and matching. Furthermore, we present an extended descriptor and criteria for real-time tracking, and evaluate the performance with both simulation and actual system.

## 1. Introduction

The estimation of target pose and position and their transitions over time is a large research area in the field of machine vision. Most non-contact motion estimation methods are based on luminance images, and in these methods, target motion between two images is estimated by using templates [1,2], interest point matching [3,4,5], or machine learning [6,7]. Machine learning methods are rapidly developing and are useful when the target is known in advance and semantic understanding is needed; however, deductive approaches are still superior when a target is unknown, rich in variation, or so dynamic that high-speed estimation is needed. In particular, many methods for interest point detection and feature extraction have been proposed and have been successfully used for accurate and fast motion estimation; however, features on luminance images have a significant disadvantage that they require textured targets, and so interest points will be scarce and inaccurate when the target has poor texture [8].

On the other hand, motion estimation methods using geometrical features have been proposed. Geometric approaches are roughly classified into point-cloud-based methods [9,10,11] and normal-map-based methods [8,12], and the distinctive features of these methods are that they do not depend on the target texture, and they are capable of estimating three-dimensional motion. Furthermore, point-cloud-based methods have the advantage of being able to handle absolute position and low-frequency components of the shape. In contrast, normal-map-based methods have the advantage that they can extract high-frequency components of the shape [13] because a representative method for surface normal measurement, called photometric stereo [14], directly computes a normal vector that is a differential value of the shape. However, conventional normal-map-based methods are still naive, and the techniques evolved in methods for dealing with luminance features have not been introduced.

In this research, we focus on the binarization of features on normal maps and propose a feature called BIFNOM that keeps the advantages of state-of-the-art features on normal maps but provides fast retrieval of corresponding interest points. Additionally, we present an extended BIFNOM descriptor and additional criteria for sequential frames, which allowed us to realize accurate and high-speed five degrees-of-freedom (DOF) estimation for continuous motion and deformation. It is difficult to estimate a translation component in depth direction from normal maps, but the other five components of rigid motion can be calculated by BIFNOM from each pair of corresponding interest points, and so BIFNOM enables estimation of denser motion and deformation. In this paper, we evaluate the computation time and accuracy of BIFNOM through a comparison with state-of-the-art feature on normal maps, and we demonstrated high-speed estimation of motion and deformation with BIFNOM accelerated by a GPU. Furthermore, we develop a vision system to measure normal maps at high speed and apply the proposed method to actual scenes. Our method can be used for real-time motion estimation regardless of whether a target is textured or not, and rigid or non-rigid. The BIFNOM with these features will be a strong tool in various applications.

## 2. Related Researches

While many kinds of features on luminance images or point clouds have been proposed, only a few features have been researched for normal maps. However, features on luminance images cannot work for textureless targets, and three dimensional point clouds are not always available when a measurement system is based on a photometric stereo method. Hence, dedicated features for normal maps are required. As conventional features on normal maps, PHENOM [8] and RIFNOM [12] are known. These conventional methods enable to define interest points, describe features and find matching of the features on normal maps. However, they do not fully use advantages of normal maps or focus only on offline processing.

For example, in interest point detection in PHENOM, which is a feature on normal maps, template patches prepared in advance were used to find pairs of corresponding interest points and calculate rotation between the pair, and the repeatability depended on the templates and implementation. Moreover, the PHENOM feature cannot be used to estimate continuous rotation even though every pair of corresponding interest points on a normal map has information about continuous three-dimensional rotation. This drawback comes from the formulation of the orientation for an interest point.

RIFNOM solved these problems in PHENOM by assigning a local coordinate system to each interest point and is capable of estimating continuous motion and deformation for each pair of corresponding interest points. Nevertheless, the performance of the RIFNOM feature has not been investigated sufficiently. Besides, the RIFNOM feature uses a heavy-weight descriptor that results in a huge amount of data and computations. As a result, it takes time to find a pair of corresponding interest points in RIFNOM. In luminance images, meanwhile, some binarized features such as BRIEF, ORB and BRISK [15,16,17] have been proposed, and these features involve less data and a lower amount of computation without significant degradation of distinctiveness. This technique will be useful also for features on normal maps.

In summary, a new feature that uses advantages of normal maps, and provides high-speed detection and light-weight descriptor is required. Furthermore, the performance evaluation and comparison with the conventional feature on normal maps need to be examined in detail. In this paper, we introduce a new binarized feature, BIFNOM based on RIFNOM and add extension of descriptor and criteria for high-speed tracking on sequential frames. Moreover, we investigate their performances through simulation-based evaluations with synthetic normal maps and applications to practical scenes with measured normal maps.

## 3. BIFNOM Features

### 3.1. Normal Maps

Normal maps can be obtained by methods including photometric stereo and differentiating depth maps. In this paper, we assume that dense surface normals are obtained as a normal map, and describe normal maps as follows:

(1) $$I_{R}\left(x,y\right)=\mathrm{round}\left(\frac{\left(n_{x}\left(x,y\right)+1.0\right)\left(2^{N_{bit}}-1\right)}{2}\right).$$

Here, $I_{R}\left(x,y\right)$ is the red intensity value of a pixel at (x,y) on a normal map with $N_{bit}\;\mathrm{bit}$ color depth, and $n_{x}\left(x,y\right)$ is the X component of a unit normal vector n(x,y) that corresponds to the surface projected to the pixel at (x,y). The value is rounded to an integer using the round function. Similarly, green and blue intensity values, $I_{G}$ and $I_{B}$, are determined by $n_{y}$ and $n_{z}$, respectively. In addition, a background region in which no object is captured is described as black, that is, $I_{R}=I_{G}=I_{B}=0$. Although there are some other color systems in visualization of surface normals, this description seems to be widely used.

### 3.2. Local Coordinate System

**Figure 1:**
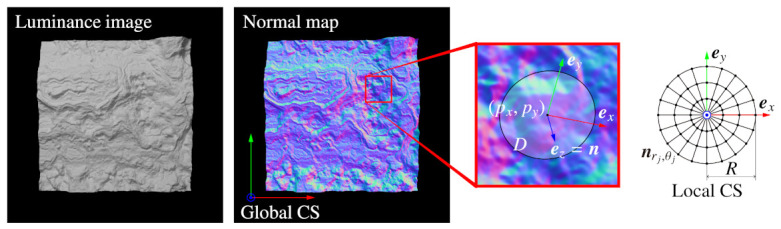
Local coordinate system (CS) assignment.

As the three-dimensional orientation of a point, we define a local coordinate system (CS) at each pixel of a normal map by using a normal at that point, n, and normals $n_{i}$ in neighbor region *D* shown in Figure 1. In most features in luminance images, orientation is defined as specifying the in-plane rotation of a point, but the orientation of BIFNOM is different from them in that it configures a local CS to specify three-dimensional rotation. Additionally, the neighbor region for determining the local CS and a feature value is defined by an ellipse, described as shown below, instead of a circle. The equation describes an ellipse with a long radius *R* at the position of the center pixel $\left(p_{x},p_{y}\right)$. The angle θ is defined by the direction of $\left(n_{x},n_{y}\right)$.

(2) $$D:\frac{{\left(x^{\prime }\;\cos\theta \;+\;y^{\prime }\;\sin\theta \right)}^{2}}{{\left(n_{z}R\right)}^{2}}+\frac{{\left(-x^{\prime }\;\;\sin\theta +y^{\prime }\;\;\cos\theta \right)}^{2}}{R^{2}}\leq 1,$$

$$\mathrm{where}\;x^{\prime }=x-p_{x},\;y^{\prime }=y-p_{y}.$$

This elliptic region *D* describes a three-dimensional circular area distorted by projection onto the image plane, and the distortion is intended for extracting almost the same region regardless of the direction of the normal vector of the center pixel, n. Although it requires that the neighbor region be flat to exactly extract the same region regardless of the direction of n, elliptic distortion in Equation (2) is reasonable because the radius *R* is small and ellipses are projected as ellipses by a perspective projection. The local CS is defined by using this *D* as follows:

(3) $$e_{x}=\frac{m}{∥m∥},\;e_{y}=e_{z}\times e_{x},\;e_{z}=n,\;$$

$$\mathrm{where}\;m=\frac{1}{N_{D}}\sum_{D}\left(n_{i}-\left(n_{i}\cdot n\right)n\right)$$

Here, $e_{x},e_{y},e_{z}$ are unit bases configuring the local CS, and $N_{D}$ is the number of pixels in the elliptic region *D*. The local Z axis is defined as the same direction as the normal of the center pixel n. The local X axis is defined by normals $n_{i}$ in the neighbor region in the Gram-Schmidt manner, and the local Y axis is defined as the cross product of $e_{z}$ and $e_{x}$.

### 3.3. Interest Point Detection

In order to find interest points whose local CS is noise-robust, we sort out pixels on a normal map with the following two criteria:

(4) $${∥m∥}^{2}>\mu_{mean},\;\frac{1}{N_{D}}\sum_{D}{∥n_{i}-m∥}^{2}>\mu_{var}.$$

The first criterion is intended to extract points that have repeatability of the local X axis. Where the norm of m is small, the direction of $e_{x}$ could be unstable against noise, and so the repeatability of the orientation will be low. Additionally, the second criterion is intended to extract points that have uniqueness for interest point matching. Where the neighbor region is almost flat and the variance of normals in the region is small, it would be difficult to find a unique pair. Hence, we set thresholds $\mu_{mean}$ and $\mu_{var}$ for the norm and variance, respectively. In addition, points around a contour that has a background area in the neighbor region are not regarded as interest points because such points are not always around the contour and have low repeatability when the target or the camera has moved. Nevertheless, we can assume that the silhouette of a target will not change significantly between two sequential frames, and so this additional criterion can be removed in this case. We mention the details of the case of sequential frames in Section 3.7.

### 3.4. Binary Feature Description

**Figure 2:**
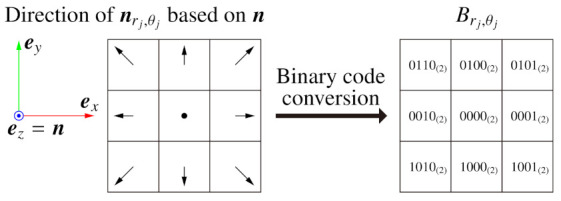
Binary codes based on the normal of the center pixel.

In BIFNOM, normals in the neighbor region are converted to binary codes and used as a feature value at the interest point. First, we divide the neighbor region into an $N_{r}\times N_{\theta }$ polar grid based on the local X axis, as shown in Figure 1. Second, we obtain a normal at each grid point $n_{r_{j},\theta_{j}}\;\left(r_{j}=1,2,\ldots ,N_{r},\;\theta_{j}=0,1,\ldots ,N_{\theta }-1\right)$ by using bilinear interpolation. Next, the interpolated normal is converted to a binary code $B_{r_{j},\theta_{j}}$ as follows (see Figure 2):

(5) $$B_{r_{j},\theta_{j}}=f\left(e_{x},\;\;n_{r_{j},\theta_{j}},\;\;2^{0}\right)+f\left(e_{y},\;\;n_{r_{j},\theta_{j}},\;\;2^{2}\right),$$

$$\mathrm{where}\;f\left(a,b,c\right)=\left\{\begin{array}{cc} 2^{0}\times c & \left(a\cdot b>+\mu_{bin}\right)\\ 2^{1}\times c & \left(a\cdot b<-\mu_{bin}\right)\\ \;0\;\;\times c & (otherwise). \end{array}\right.$$

In this conversion, a normal is classified into 9 groups, while some methods for luminance images classify the luminance value into 2 or 3 groups [15,16,17,18]. The binary codes are set to describe which direction the neighbor normal $n_{r_{j},\theta_{j}}$ points in based on the normal of the center pixel n and form the $L_{1}$ space in the table with the Hamming distance. Although a 4bit code can describe at most $2^{4}=16$ groups, we designed the classification with an odd-times-odd table to robustly classify a normal similar to n into the center cell. Finally, we obtain the BIFNOM feature vector $B=(B_{1,0},\ldots ,B_{N_{r},N_{\theta }-1})$ which consists of $4N_{r}N_{\theta }\;\mathrm{bit}$.

In RIFNOM, local X and Y components of normals in the neighbor region are stored in the feature, and so when 4byte is used for floating-point data, the amount of data for a single normal in the neighbor region is 8bytes=64bits in RIFNOM compared with 4bits in BIFNOM. The influence of data compression will be investigated in Section 4.

### 3.5. Interest Point Matching

To estimate the motion and deformation of a target, we compare a set of BIFNOM features extracted in a reference frame to those extracted in another frame, and find pairs of corresponding interest points. The distance between two BIFNOM features is defined by the sum of the Hamming distances. Here, let $H_{min1}$ and $H_{min2}$ be respectively the minimum distance and second-minimum distance that were found by the comparison between an interest point in the reference frame and all features in the other frame. Then, a unique match is determined by a pair of corresponding interest points satisfying the following similarity test and ratio test.

(6) $$H_{min1}<\mu_{diff},\;\frac{H_{min1}}{H_{min2}}<\mu_{ratio}.$$

This matching method uses a brute-force comparison in which the distances for all combinations are calculated but the retrieval would be more simple when the reference frame and the other frame are sequential. We describe this special condition in Section 3.7.

### 3.6. Estimation of Motion and Deformation

With the proposed method, 5 DOF motion, including two-dimensional translation in the image plane and three-dimensional rotation, can be estimated from each pair of corresponding interest points by assigning the local CS to each interest point. As a result, BIFNOM can estimate higher-DOF and denser motion and deformation than features on luminance images.

With a pair of corresponding interest points, A and B, the translation vector T and the rotation matrix *R* between the points are estimated as follows:

(7) $$T=p_{B}-p_{A},\;R=E_{B}E_{A}^{-1},\;$$

$$\mathrm{where}\;p={\left(p_{x}\;\;p_{y}\right)}^{T},\;E=\left(e_{x}\;\;e_{y}\;\;e_{z}\right).$$

Here, p and *E* are the position and local bases of an interest point, respectively, and the indexes A and B denote the point to which a variable belongs. The translation T is derived from the difference of the positions, and the rotation *R* is derived from the transformation between the local CSs.

### 3.7. Extensions for Sequential Frames

If there is an assumption that input images are sequential and motion and deformation in the interval are small, we can extend the BIFNOM descriptor and the criteria to improve the number of interest points and the accuracy of the matching. First, we can remove the ratio test in the Equation (6).

Furthermore, we can assign a binary code also to the background area. In the interest point detection for general images, we exclude points whose neighbor region includes the background area. On the other hand, in the extension for sequential frames, we detect interest points regardless of whether the background is included since the background area in a certain frame will not be significantly changed in the next frame. In our implementation, we use a code $1111_{\left(2\right)}$ for the background in consideration of the Hamming distance, against the other binary codes indicated in Equation (5) and Figure 2.

Finally, we can use an additional criterion for interest point matching. When the two input frames are sequential, the translation of an interest point during the interval will be small:

(8) $$∥p_{A}-p_{B}∥<\mu_{track}.$$

Here, $\mu_{track}$ represents a search range for interest point matching. Hereafter, we refer to the extended BIFNOM described in this subsection as “BIFNOM for tracking”.

## 4. Evaluation Experiments

### 4.1. Advantages and Experimental Setup

The advantages of BIFNOM include high detection ability of interest points on textureless targets and the high-speed estimation ability of 5 DOF motion and deformation. Accordingly, we evaluated the performance in the case of a textureless target, in term of the computation time and the accuracy. In all subsequent experiments, we experimentally set the parameters to R=15, $N_{r}=3$, $N_{\theta }=20$, $\mu_{mean}=0.15$, $\mu_{var}=0.25$, $\mu_{bin}=0.25$, $\mu_{diff}=15$, $\mu_{ratio}=0.63$, and $\mu_{track}=40$ except where specifically noted.

### 4.2. Textureless Target

**Figure 3:**
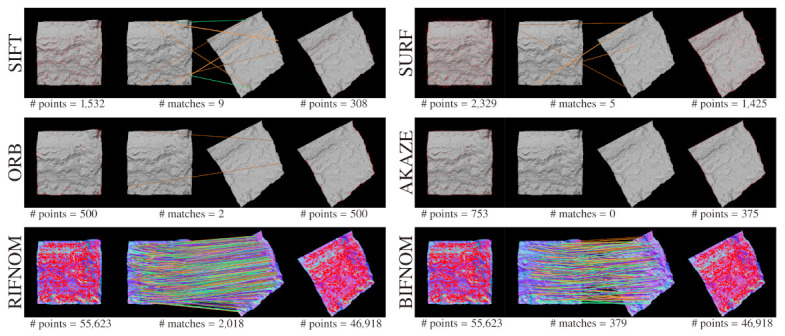
Interest point detection and matching for a textureless target. Detected interest points are shown in red. The symbol “#” means the number of interest points or matches.

First of all, we show the advantages of features on normal maps as compared with features on luminance images with a textureless target. Figure 3 shows the results of interest point detection and matching on luminance images and normal maps. In the figure, the target is the same but the number of interest points detected by BIFNOM or RIFNOM [12] is larger than SIFT [3], SURF [4], ORB [16] and AKAZE [5]. Moreover, the accuracy of the matching with features on normal maps also seems to be higher than with features on luminance images. For textureless targets, the performance of features on normal maps is apparently and logically superior to features on luminance images and this single evaluation is enough to show the advantage. The difference in the number of matches of two normal-map-based features comes from the definition of the distance and the thresholds used. In RIFNOM, the distance between features are calculated with inner product between unit vectors, and each product results in a continuous value within [−1.0,1.0]. On the other hand, the distance in BIFNOM is defined as Hamming distance between binarized 4 bit codes as shown in Section 3.4 and each distance derives a discrete value within [0,4]. This difference does not show the detection performance and they can be easily adjusted by changing $\mu_{diff}$ and $\mu_{ratio}$. The estimation of three-dimensional rotation and a quantitative evaluation are described in Section 4.4.

### 4.3. Computation Time

As mentioned in Section 3.4, the amount of data for the BIFNOM descriptor is one-sixteenth of that for RIFNOM with the same $N_{r}$ and $N_{\theta }$. This reduction in the amount of data also contributes to a reduced amount of computation and lower computation time. The computation time for a 640×480 VGA-size sequential images is shown in Table 1. In this evaluation, RIFNOM, BIFNOM are calculated in parallel on a GPU (GPU:nVidia Quadro P2000, Titan V, CPU:Intel Xeon Gold 5122 3.60GHz 2 Processors, Memory:32GB), and the computation time is obtained from 360 frames. The result shows that total time of interest point detection and matching with the BIFNOM feature is at least 4-times faster than that with the RIFNOM feature and can be completed in about 1.3ms. As a result, real-time estimation of motion and deformation even for VGA 750fps video can be realized by using the BIFNOM features.

### 4.4. Accuracy

**Figure 4:**
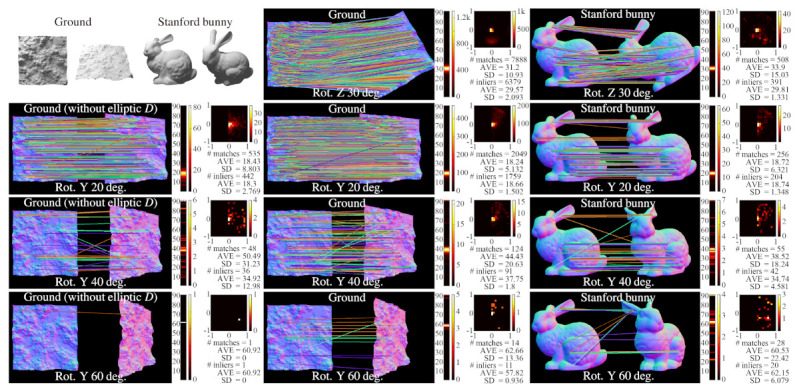
Estimated motion between images of a rigid body model, “Ground” or “Stanford bunny”, and the rotated one, using BIFNOM. For each case, a histogram and statistics for estimated rotation angles and a heat map for estimated rotation axes are described. In the heat map, XY components of a unit direction vector of the rotation axis are shown for the rotation around the Z axis, and XZ components are shown for the rotation around the Y axis, and so the ground truth will appear as a center spot. The first column shows the importance of the elliptic region *D*.

Figure 4 shows the results of motion estimation by using BIFNOM for rotated rigid body targets. The lines connecting corresponding interest points show the plausibility of estimation of translation. Additionally, the histograms of estimated rotation angle and heat maps of estimated rotation axes show the accuracy of estimated three-dimensional rotation. With a large inclination angle, the accuracy of the motion estimation is degraded mainly by occlusion; however, results using a circular neighbor region instead of the elliptic region *D* show that the elliptic region *D* prevents the performance from significantly deteriorating.

**Figure 5:**
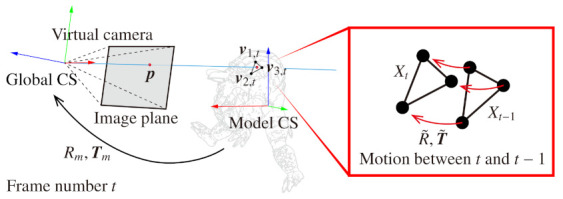
Derivation of the ground truth of motion and deformation.

Furthermore, we also quantitatively evaluated the accuracy of the estimation of motion and deformation in sequential frames. We rendered sequential images by non-rigid body simulation [19] and prepared test sequence and ground truth. In this simulation, a 3D model “Armadillo” was moved and deformed according to gravity and user interaction, and the ground truth of the motion and deformation was derived based on $X_{t}=\left(\;v_{1,t}\;\;v_{2,t}\;\;v_{3,t}\;\right)$. The vectors $v_{1,t}$, $v_{2,t}$ and $v_{3,t}$ are the three-dimensional positions of three vertices that form a triangle mesh corresponding to a pixel of the rendered image whose frame number is *t*, as shown in Figure 5. Since all meshes of the 3D model were triangulated in advance, the number of vertices is always three. Besides, $v_{1,t-1}$ denotes the vertex $v_{1,t}$ in the previous frame.

With $X_{t}$, the ground truth of the translation $\tilde{T}$ is derived for each pixel from the difference of projected points in sequential frames as follows:

(9) $$\tilde{T}={\tilde{p}}_{t}-{\tilde{p}}_{t-1}$$

$$\mathrm{where}\;s_{t}\left(\begin{array}{c} \;\;{\tilde{p}}_{t}\;\;\\ \;\;1\;\; \end{array}\right)=PR_{m}^{-1}\left(C_{t}-T_{m}\right),\;C_{t}=\frac{1}{3}X_{t}1.$$

In the equation, 1 is a column vector with ones in all elements, and $C_{t}$ is a centroid position of the vertices $v_{1,t}$, $v_{2,t}$ and $v_{3,t}$. The centroid position $C_{t}$ is transformed by model-view parameters $R_{m}$ and $T_{m}$ to be rendered from a virtual camera, and projected to a 2D position ${\tilde{p}}_{t}$ on the rendered image by perspective projection. The matrix *P* is a projection matrix, and $s_{t}$ is a variable for the projection.

Moreover, the ground truth of the rotation $\tilde{R}$ is derived for each pixel as follows:

(10) $$\tilde{R}=V\;\mathrm{diag}(1,1,|VU^{T}\left|\right)\;U^{T},$$

$$\mathrm{where}\;USV^{T}\;\;=R_{m}^{-1}X_{t}^{\prime }{\left(R_{m}^{-1}X_{t-1}^{\prime }\right)}^{T}\;,\;X_{t}^{\prime }=X_{t}-C_{t}1^{T}$$

Here, $X_{t}^{\prime }$ is a matrix that contains vertex positions in barycentric coordinates. The positions are transformed using the virtual camera coordinates, and the product of matrices is decomposed by SVD. The ground truth of the 3D rotation $\tilde{R}$ is defined as the product of *V* and $U^{T}$ with determinant normalization. The matrix $\tilde{R}$ obtained by the above operations minimizes $\Sigma_{i}{∥\left(v_{i,t}-C_{t}\right)-\tilde{R}\left(v_{i,t-1}-C_{t-1}\right)∥}^{2}$ [20,21], and this is appropriate for the ground truth.

Additionally, we define the error of translation $T_{e}$ and error of rotation $R_{e}$ and $A_{e}$ as follows:

(11) $$T_{e}=\frac{1}{N_{t}}\sum_{i}\frac{∥T_{i}-\tilde{T_{i}}∥}{∥\tilde{T_{i}}∥}$$

(12) $$R_{e}=\frac{1}{N_{t}}\sum_{i}\frac{∥\theta_{i}-{\tilde{\theta }}_{i}∥}{∥{\tilde{\theta }}_{i}∥}$$

(13) $$A_{e}=∥\frac{1}{N_{t}}\sum_{i}\left(l_{i}-\tilde{l_{i}}\right).∥$$

The variables θ and $\tilde{\theta }$ are the estimated rotation angle and the ground truth, and l and $\tilde{l}$ are unit direction vectors of the estimated rotation axis and the ground truth. Both the rotation angle and the rotation axis are obtained by Rodrigues’ rotation formula. The errors of all $N_{t}$ pairs obtained in a frame *t* are averaged for each frame.

**Figure 6:**
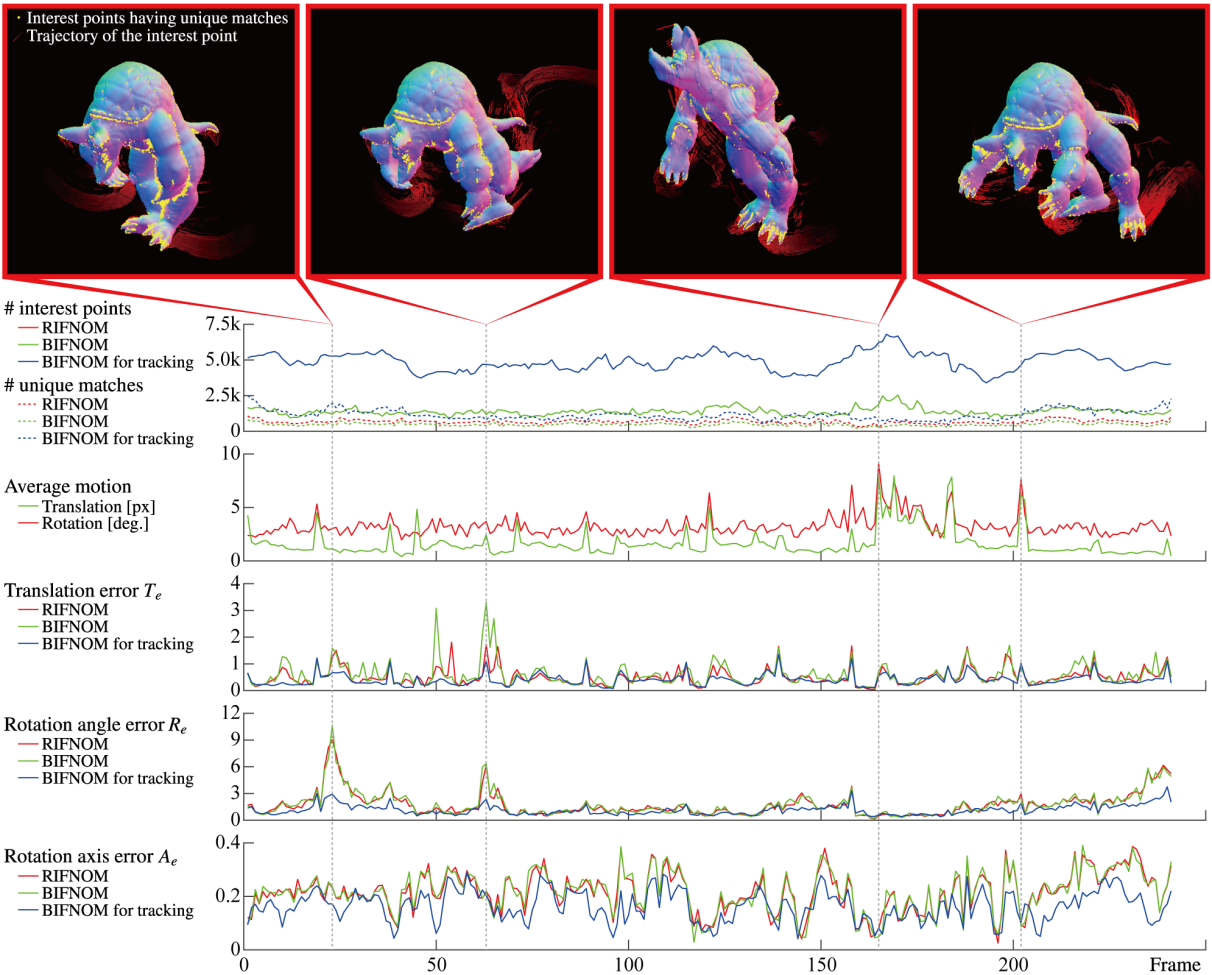
Estimated motion from sequential images of a non-rigid body model, “Armadillo”. The graph of the number of interest points for RIFNOM is the same as for BIFNOM.

Finally, estimation results for motion and deformation are shown in Figure 6. Since the detection method is the same, the number of interest points of BIFNOM was equal to that of RIFNOM. On the other hand, BIFNOM for tracking can detect more interest points thanks to adding the background code mentioned in Section 3.7. Moreover, BIFNOM can find as many unique matches as RIFNOM and the graphs show the trend over frame was also similar. By assuming continuous motion with the Equation (8) and the background code, the number of unique matches was always larger than those of BIFNOM and RIFNOM. Besides, the figure shows that BIFNOM realized an estimation accuracy on the same order as RIFNOM. The estimated motion error of BIFNOM and RIFNOM can be spiky especially when the average motion was large but BIFNOM for tracking suppressed the error to be smaller. This arises from assumption of continuous motion. The estimation error of rotation is not small because the quantization error in the Equation (1) will affect minute rotation in sequential frames; however, Figure 4 shows good performance in the general case, and the estimation can be improved by statistical processes using neighbor matches. Denser motion estimation from each match will contribute to improving performance. In summary, BIFNOM was successfully extended to a light-weight feature based on RIFNOM without serious deterioration. Moreover, BIFNOM for tracking provided better results in sequential frames. Note that Figure 6 shows only a few frames among 241 frames; see the Supplementary Materials for the details of the whole sequence.

## 5. Applications to Real Scenes

High-speed normal measurement is capable of capturing detailed surface of dynamically moving objects and useful for 3D shape reconstruction, surface inspection, and tracking for SLAM and AR/VR/MR [22]. In this section, we demonstrate tracking of objects with BIFNOM features on normal maps measured by high-speed normal measurement system.

**Figure 7:**
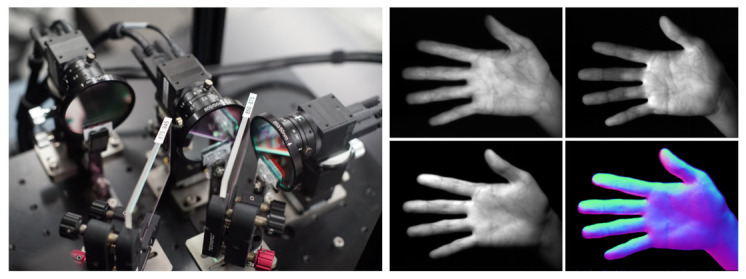
High-speed normal measurement system and normal map calculation by photometric stereo method.

**Figure 8:**
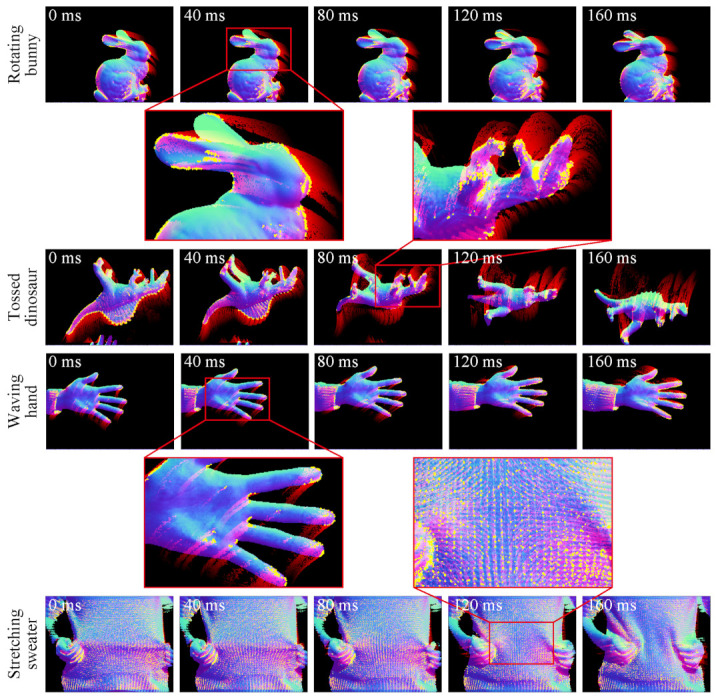
Motion tracking in actual scenes. From top to bottom, figures show scenes of rotating bunny, tossed dinosaur, waving hand and stretching sweater. Yellow dots and red fading strokes respectively show interest point having unique matches and trajectories of the interest points as Figure 6.

Figure 7 shows the high-speed normal measurement system that measures surface normal at 500fps by using wavelength-division photometric stereo method in infrared region. The system is composed of three high-speed cameras (XIMEA, MQ003MG-CM, Münster, Germany), two dichroic mirrors, and three IR LED arrays (810nm,850nm, and 940nm). An image in each IR wavelength is split by the dichroic mirrors and captured by each high-speed camera as 648×488px monochrome image in synchronization, and a normal map is calculated from these images as shown in Figure 7. By applying BIFNOM for tracking to the measured normal maps, we obtained motions of the target as shown in Figure 8.

In all scenes in the figure, the proposed method detected and tracked interest points on normal maps in the same way on synthetic data. The interest points were detected mainly on bumpy surface or contours and this indicates the criteria in Equation (4) and the binary code extension for background worked as intended. Moreover, even when a target is non-rigid and includes unidentifiable repeated pattern like stitches in the stretching sweater scene, the proposed method succeeded in finding the correct pairs thanks to the criterion in Equation (8) and abolition of ratio test. However, some misleading interest points were detected and tracked where cast shadow occurred and correct normal vector cannot be obtained as the back ear shown in rotating bunny scene at 120ms. This deterioration arises from sensing, not the proposed method, and such problems can be improved by applying more sophisticated photometric stereo methods or a setup with more redundant lighting.

## 6. Conclusions

In this paper, we proposed a novel feature on normal maps, called BIFNOM, that has the advantages that it enables fast 5 DOF motion estimation without target textures. We additionally proposed an extended descriptor and criterion for sequential frames and realized accurate estimation of motion and deformation. BIFNOM reduced the computation time by using binary codes and achieved real-time interest point detection and matching at high speed (over 750fps) and it can be applied to real-time object recognition and tracking for SLAM, AR/VR/MR and so on. Future work will include introducing a scale space for 6 DOF motion estimation including depth and application to pose and position estimation of a dynamic target for dynamic projection mapping in which features on luminance images could be unstable due to the projection. 

## Figures and Tables

**Table 1:** Computation time for interest point detection and matching. The entries, “AVE” and “SD”, are average and standard deviation, respectively.

Feature (GPU)	Detection AVE (SD)	Matching AVE (SD)
RIFNOM (P2000)	23.489 (2.570)ms	5.127 (1.258)ms
RIFNOM (TitanV)	7.425 (0.209)ms	1.442 (0.206)ms
BIFNOM (P2000)	5.942 (0.757)ms	0.665 (0.144)ms
BIFNOM (TitanV)	1.020 (0.092)ms	0.259 (0.054)ms

## Supplementary Materials

The following are available at https://www.mdpi.com/article/10.3390/s21103469/s1, Video S1: BIFNOM for tracking and the details of the whole sequence.
